# Effects of Post-Heat Treatment on Mechanical and Tribological Properties of 3D-Printed PLA and PEEK Structures

**DOI:** 10.3390/polym18020253

**Published:** 2026-01-16

**Authors:** Yunxiang Deng, Li Chang

**Affiliations:** School of Aerospace, Mechanical and Mechatronic Engineering, The University of Sydney, Sydney, NSW 2006, Australia; yden8342@uni.sydney.edu.au

**Keywords:** post-heat treatment, re-entrant structure, high-performance polymer, additive manufacturing, friction and wear

## Abstract

In the present study, post-heat treatment was applied to improve the mechanical and tribological performance of 3D-printed polymer components. Two polymers, i.e., polylactic acid (PLA) and polyether ether ketone (PEEK), were used as base materials. Re-entrant structures were incorporated into printed specimens to mitigate friction-induced vibrations (FIV). The results showed that the heat-treatment process effectively enhanced the mechanical properties of both materials by increasing their elastic modulus and yield strength. Specifically, the tensile and compressive strengths of heat-treated PLA increased from 44.14 MPa to 47.66 MPa and from 68 MPa to 82 MPa, respectively. A similar trend was observed for heat-treated PEEK, with tensile strength increasing from 75.53 MPa to 84.91 MPa and compressive strength from 106 MPa to 123 MPa. Furthermore, the increased stiffness enabled the re-entrant structures to more effectively reduce FIV during the sliding process of specimens. However, heat treatment produced contrasting effects on the wear performance of the two polymers. The specific wear rate of the heat-treated PLA sample with the re-entrant structure increased from 2.36 × 10^−5^ mm^3^/(N · m) to 4.5 × 10^−4^ mm^3^/(N · m), while it decreased for the PEEK sample from 3.18 × 10^−6^ mm^3^/(N · m) to 6.2 × 10^−7^ mm^3^/(N · m). Microscopic observations revealed that this difference was due to the variations in the brittleness of the treated materials, which influenced wear-debris formation and the development of the transfer film on the steel counterface. These findings demonstrate that post-heat treatment is an effective method for tailoring and optimizing the mechanical behavior of printed polymers while also emphasizing the necessity of systematically evaluating its influence on the tribological performance of printed engineering parts subjected to different sliding conditions.

## 1. Introduction

Over the past decade, there have been rapid advancements in additive manufacturing, which enable the creation of complex 3D components by adding material layer by layer, using a wide range of engineering materials such as polymers, metals, ceramics, and concrete [1,2]. In particular, the production of polymeric parts using fused deposition modeling (FDM) has gained significant attention in the field, owing to its design flexibility, low cost, and time-efficient prototyping capabilities [3].

The most common polymers used in FDM technology include acrylonitrile butadiene styrene (ABS), polylactic acid (PLA), and polycarbonates (PC), etc. [4,5,6]. Among these, PLA filament stands out as the most widely used material in 3D printing due to its low cost, ease of processing, biodegradability, and biocompatibility [6]. These attributes make PLA attractive for a variety of applications, particularly in the biomedical field [7]. Nevertheless, PLA exhibits limitations that restrict its broader engineering applications, such as low toughness and poor heat resistance [6].

Recently, the processing of high-performance polymers (HPPs), such as polyimide (PI), polyetherimide (PEI), and polyether ether ketone (PEEK), through 3D printing has attracted significant attention [8,9,10,11,12]. In particular, PEEK has been extensively studied due to its outstanding physicochemical properties, including excellent heat resistance, mechanical strength, corrosion resistance, biocompatibility [13,14,15,16], and high wear resistance [17]. These qualities make PEEK an ideal material for a wide range of applications, such as aerospace, medical, and tribological systems [18,19,20,21]. However, the fabrication of PEEK via fused filament fabrication (FFF) presents several challenges. For instance, due to the relatively high printing temperature required, thermal residual stress can accumulate, leading to distortion and interlayer delamination [9]. Consequently, 3D-printed PEEK parts often exhibit inferior mechanical properties compared to conventionally injection-molded PEEK, primarily due to printing-induced defects such as weak interlayer bonding, distortion, and delamination [10,11]. To address these limitations, extensive research has focused on optimizing FDM process parameters for PEEK [10,11]. Wang et al. employed finite element analysis (FEA) to simulate the melting behavior and fluidity of PEEK to identify suitable printing parameters for achieving adequate surface quality and enhanced mechanical performance [11]. Their study concluded that a higher nozzle temperature of 440 °C and a reduced layer thickness of 0.1 mm improved part density, reduced internal defects, and strengthened interlayer and infill bonding. Tafaoli-Masoule et al. applied a Taguchi design of experiments (DoE) approach to systematically investigate the influence of FDM parameters such as nozzle temperature, bed temperature, print speed, and layer thickness on the tensile strength and elongation at break of printed materials [10]. Rosa-Sainz et al. [22] investigated the use of PEEK for customized cranio-maxillofacial prostheses, focusing on its experimental formability. The results showed that, under optimized processing conditions, thermoformed patient-specific shapes derived from 3D-printed PEEK sheets exhibited excellent thermoformability and dimensional accuracy. Feng et al. [23] explored the application of machine learning (ML) techniques to optimize PEEK printing parameters in FDM processes. Their findings demonstrated that comprehensive training on the effects of multiple critical printing parameters enabled the ML algorithm to effectively identify optimal printing conditions for enhanced material performance.

Nevertheless, despite these advances, fully overcoming the intrinsic defects associated with the FDM processing of HPPs remains challenging. The high melting temperatures of HPPs, combined with natural cooling and thermal gradients during printing, inevitably compromise the degree of crystalline perfection in printed parts, thereby adversely affecting their mechanical properties. As a result, post-processing treatments have become a necessary step to enhance the structural integrity and overall performance of printed polymer components [24]. Up to now, various post-treatment methods have been developed to improve the performance of FDM-printed components [25]. For instance, Li et al. [26] investigated the chemical treatment, such as the hot vapor chemical smoothing, on the surface roughness of the printed PLA parts. The result was promising, showing a significant reduction in surface roughness of the 3D-printed PLA components through the optimized Taguchi L16 experimental design. Pricci et al. [27] studied the experimentally validated digital twin of the ironing process in extrusion-based additive manufacturing. The result demonstrated that the optimized ironing parameters can remarkably enhance tensile strength. In particular, the combination of ironing temperature, pressure, and speed improved the ultimate tensile strength by improving the layer bonding and reducing surface defects. Moradi et al. [28] investigated the effect of the utilization of CO_2_ laser beam cutting as a post-processing technique on the dimensional accuracy and edge quality of the FDM-printed PLA parts. The results demonstrated that the optimized laser parameters can significantly improve dimensional accuracy by reducing the kerf width and taper and producing high-quality cuts.

Among the various post-processing techniques, post-heat treatment (or annealing) has emerged as one of the most important methods for improving the mechanical properties of printed parts [29]. The post-heat treatment utilized the temperature changes to expand and activate the movement of the polymer molecular chain, facilitating the recrystallization of crystalline and semi-crystalline polymers [30,31]. In addition, while relieving the residual stress during the heat treatment process, the interlayer bonding was improved to minimize the internal FDM 3D-printed defects [32,33,34]. For instance, Yang et al. [30] investigated the effects of different thermal processing conditions during 3D printing on the degree of crystallinity and mechanical performance of neat polyether ether ketone (PEEK). Their findings revealed that optimizing the ambient temperature, nozzle temperature, and post-heat treatment methods could increase crystallinity and thus enhance material strength and stiffness. Kumar et al. [35] studied the combined effects of infill density and annealing on the mechanical performance of a selected filament. Their results demonstrated that samples with higher infill density showed improved tensile strength and stiffness after post-annealing. Zhen et al. [36] examined the influence of 3D printing parameters and annealing conditions on the microstructure and mechanical performance of printed PEEK. It was found that annealing or post-heat treatment improved tensile strength, ductility, and overall structural integrity due to enhanced crystallinity, interlayer adhesion, and reduced residual stress. Dong et al. [37] analyzed the effects of post-processing annealing on 3D-printed nanofiber-reinforced polylactic acid (PLA). Their study highlighted that annealing promoted increased flexural strength and modulus, owing to improved polymer crystallization and strengthened fiber-matrix interfacial adhesion.

Despite the beneficial effects of post-heat treatment on mechanical performance, its impact on the time-dependent tribological performance of materials, particularly in complex structures, remains limited [38,39]. While it is often assumed that improvements in mechanical properties lead to enhanced tribological performance, tribological properties are not intrinsic material properties and instead depend strongly on the specific system and operating conditions in which a material or structure functions. This study aims to investigate the effects of post-heat treatment on the mechanical and tribological performance of FDM-printed engineering structures. In our previous work, we explored the use of re-entrant auxetic structures via 3D printing as novel self-tuning absorbers for mitigating friction-induced vibrations (FIV) [40]. The study demonstrated the potential of the re-entrant structure in suppressing FIV of neat PLA sliding against steel counterparts. However, stress concentrations and printing defects in the printed structures can compromise energy absorption efficiency and load-bearing capacity. In the present study, for the first time, post-heat treatment is applied to complex tribological components incorporating embedded re-entrant auxetic structures fabricated using FDM. Annealing was selected as a cost-efficient post-treatment method that does not require the high capital investment or expensive consumables associated with other techniques. More importantly, compared with post-processing methods such as chemical smoothing, annealing was shown to improve the overall material properties of the entire volume of the printed part rather than being limited to surface refinement. The influence of the proposed heat treatment on the mechanical and tribological properties of the printed base materials and structures was systematically investigated. This work contributes to the advancement of heat treatment processes for thermoplastics, offering guidance for their application in a wide range of tribological applications and leveraging the capabilities of modern additive manufacturing techniques.

The structure of this paper is as follows: after Section 1, Section 2 describes the materials preparation process and the related experimental setup, including the post-heat treatment method, mechanical testing, and tribological test configuration; Section 3 presents the experimental results, specifically the effects of post-heat treatment on the mechanical and tribological properties of the printed parts; and Section 4 summarizes the main findings and discusses the limitations of the current work.

## 2. Materials and Experimental

### 2.1. Materials Preparation

All samples were made by Fused Deposition Modeling (FDM). The printer used for printing the neat PLA was the Prusa CORE One, manufactured by Prusa Research, a.s., Prague, Czech Republic (EU), and the neat PEEK was printed with the FUNMAT HT made by INTAMSYS, Technology Co., Ltd., Shanghai, China. The selected neat PLA was manufactured by eSUN, Shenzhen Esun Industrial Co., Ltd., Shenzhen, China, and KetaSpire PEEK MS NT1 was made by Solvay, Brussels, Belgium. The diameter of both filaments is 1.75 mm. All specimens were printed in solid infill with different orientations. The infill angle of 90 degrees was employed for material testing. The printing direction of the re-entrant auxetic wear model was controlled along the structure direction to maximize the loading capacity of the auxetic structure [41].

The printing parameters for the two filaments, including nozzle temperature, bed temperature, and chamber temperature, were determined based on our previous studies and the manufacturer’s recommendations [40,42], as listed in Table 1. In addition, a layer height of 0.2 mm and a printing speed of 25 mm/s were used for both filaments.

### 2.2. Characterization of Mechanical Behavior

**Figure 1:**
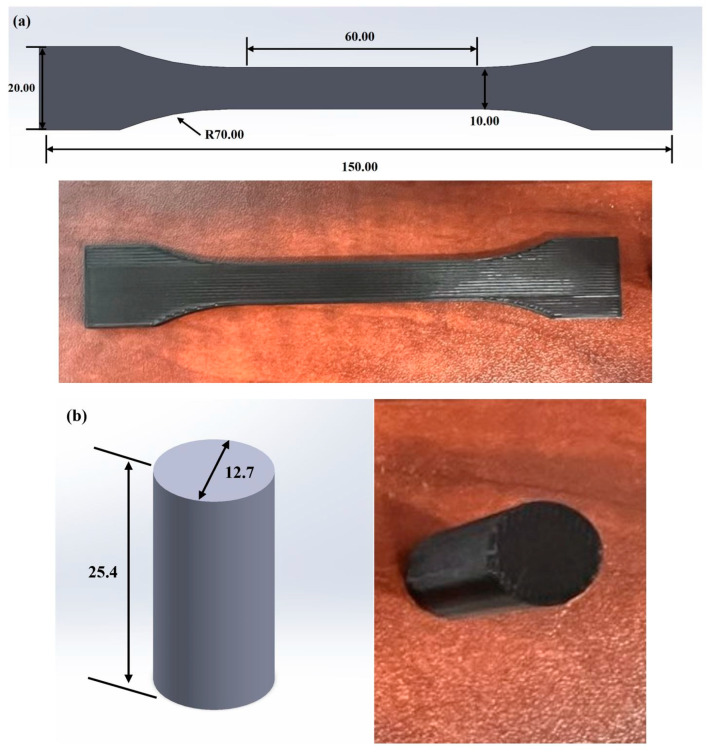
Schematic: (**a**) tensile test specimen and (**b**) compression test specimen. Dimensions are indicated on the figure in millimeters (mm).

The mechanical properties of tensile strength were examined using the Instron 3366 Universal Testing machine following the ISO 527-1 standard [43] at a test speed of 50 mm/min. The compressive strength of samples was evaluated by the Instron 5567 Universal Testing machine, following the standard of ASTM D695 [44] at a rate of 1.3 mm/min. Figure 1a,b shows the design and printed specimens for tensile and compression tests, respectively.

Dynamic mechanical analysis (DMA) was conducted on a Discovery DMA 850, manufactured by TA Instruments, New Castle, DE, USA, to determine the T_g_ (glass transition temperature) of the filament. The sample was tested under an amplitude of 20 μm at a frequency of 1.0 Hz. The temperature was ramped from 35 °C to 80 °C for neat PLA and from 35 °C to 250 °C for neat PEEK. The same ramp rate of 3.0 °C/min was applied to both filaments.

Differential scanning calorimetry (DSC) was performed on DSC 2500, manufactured by TA Instruments. The degree of crystallinity of the PEEK sample with and without the post-heat treatment was calculated using the following equation:

(1) $$X_{c}=\frac{{∆H}_{m}}{{∆H}_{m}^{0}}\times 100\%$$

where ${∆H}_{m}$ is the enthalpy of melting obtained by the heat flow curve after the baseline correction, and ${∆H}_{m}^{0}$ is the enthalpy of fully crystalline PEEK, which is 130 J/g.

The temperature setting and duration of the standard PEEK and the heat-treated PEEK (PEEK-HT) are identical, as shown in Table 2.

### 2.3. Post-Heat Treatment

The post-heat treatment was conducted with the furnace, Nabertherm TR60 oven, manufactured by Nabertherm GmbH, Lilienthal, Germany. The treatment time of neat PLA and PEEK was summarized in Table 3. The duration and temperature of the specific post-heat for PLA and PEEK were decided in accordance with the thermal properties. For PLA, a single-step heat treatment above the T_g_ was utilized to relieve the residual stress and enhance the crystallinity following established approaches in the literature [42], while considering a relatively low melting temperature typically around 170–180 °C. For PEEK, which has a significantly higher T_g_ at 143 °C and melting temperature at 343 °C, a multi-stage heat treatment was applied based on prior studies and manufacturing guidelines [30,36,45,46,47]. This approach was intended to optimize crystallization and mechanical properties by gradually relieving residual stress and enhancing material crystallinity. In addition, the incremental temperature increases from 200 °C to 220 °C helped to control stress relaxation and avoid potential thermal shock associated with rapid temperature elevation.

### 2.4. Friction and Wear Tests

**Figure 2:**
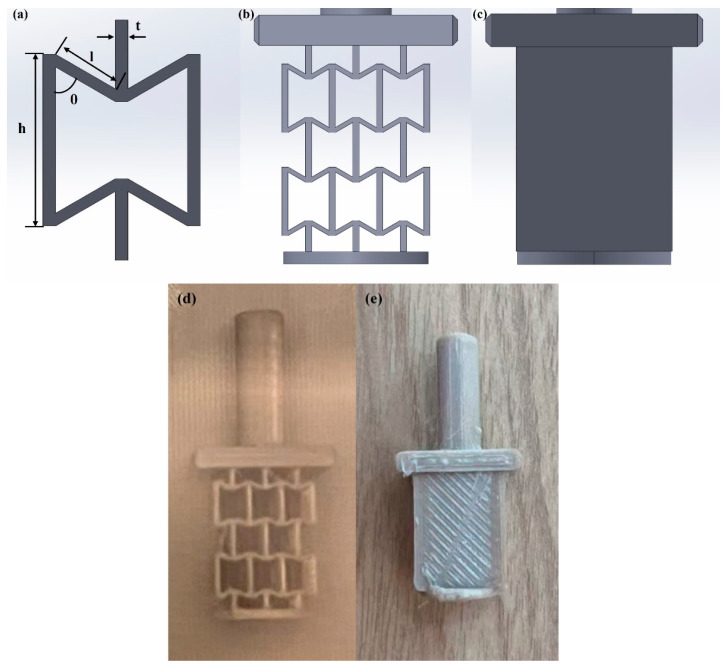
Schematics: (**a**) re-entrant auxetic unit with the re-entrant angle of 60°, (**b**) the sample with the re-entrant auxetic structure (Re60), (**c**) the solid benchmark sample for wear tests, (**d**) the printed sample with the re-entrant auxetic structure, and (**e**) the printed sample without the re-entrant auxetic structure.

The design of the wear specimens was followed by our previous study [40]. Figure 2a shows the design of a single unit cell for the re-entrant auxetic structure, which can be defined by four parameters: height (h), length (l), angle (θ), and thickness (t) [48,49]. Figure 2b demonstrates the re-entrant wear test model assembly with unit cells, and Figure 2c shows the comparison wear model, the solid benchmark in which the re-entrant auxetic structure is represented by a solid rectangle.

**Figure 3:**
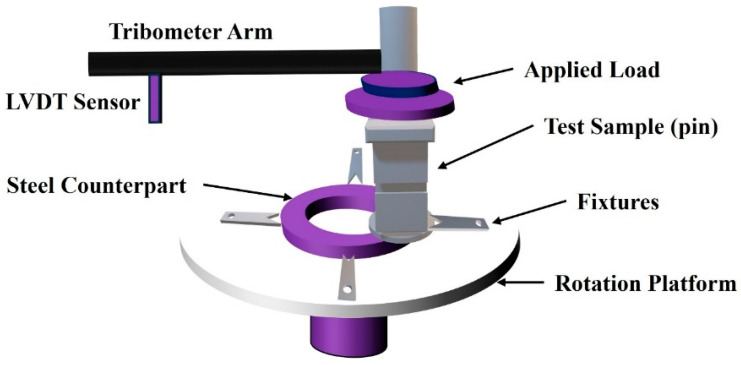
Schematic of the pin-on-disk wear-test machine and setup.

As shown in Figure 3, the friction and wear tests were conducted at room temperature using a pin-on-disk configuration with a commercial tribometer NANOVEA-MT/60/NI (Nanovea, Irvine, CA, USA). The counter body was a stainless-steel disk (SKF Gothenburg, Sweden; model LS2542) with an internal diameter of 25 mm and an external diameter of 42 mm. The steel counterpart had a hardness of approximately 910 HV and a surface roughness of about 220 nm. Each test was run for a total of 600/900 min at a constant disk rotation speed of 120 rpm with an applied normal load of 5 N for PLA and 25 N for PEEK.

The specific wear rate (SWR) of the testing specimen was calculated based on the mass loss during the test in the formula below [50]:

(2) $$SWR=\frac{∆m}{\rho \cdot F_{N}\cdot L}$$

where ∆m denotes mass loss, ρ is the material density, $F_{N}$ is the normal force, and L is the sliding distance.

**Figure 4:**
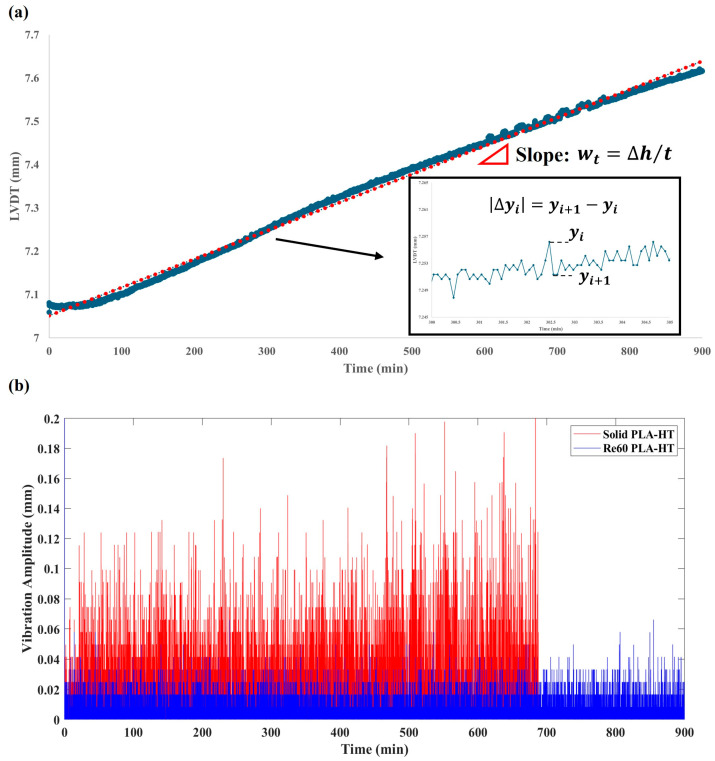
Vibration amplitude (**a**) LVDT results; a linear trend line (the “dotted line linear fitting”) has been applied to data points and the slope of this fitted line then quantifies the rate of wear height loss per unit of time; (**b**) derived vibration amplitude curve for PLA-HT (heat treated) solid benchmark (Solid PLA-HT) and PLA with the embedded re-entrant auxetic structure (Re60 PLA-HT) at the load of 5 N.

To measure friction-induced vibration in the vertical direction, the linear variable differential transformer (LVDT) sensor was mounted on the tribometer arm to track the vertical displacement of the sample. Figure 4a illustrates a representative curve of height variation as a function of sliding time captured by the LVDT sensor. The curve represents the wear re-entrant auxetic model at a 60-degree angle, printed with PLA. The displacement data generally increases due to material wear loss, leading to a decrease in sample height. The slope of the curve indicates the time-dependent wear rate, $w_{t}=∆h/t$, where ∆h is the sample height loss and t denotes time. To analyze the friction-induced vibration separately from wear, the absolute value of the immediate displacement change $\left|∆y_{i}\right|$ was used to determine the vibration amplitude in millimeters. The assumption is that over very short time intervals, wear is negligible; therefore, fluctuations in displacement during these intervals primarily reflect the system’s vibration behavior. The average vibration amplitude is calculated using the equation below:

(3) $$AV=\frac{\sum_{i=1}^{n}\left|∆y_{i}\right|}{n-1}=\frac{\sum_{i=1}^{n}\left|\left(y_{i+1}-y_{i}\right)\right|}{n-1}$$

where y is the measured displacement between the LVDT sensor and the sample.

Through the equation, the corresponding vibration amplitude curve derived from Figure 4a is shown in Figure 4b, and the result of the average vibration amplitude is 0.00111 mm. For comparison, the average vibration amplitude of the solid benchmark is also given in Figure 4b, with a value of vibration amplitude equal to 0.00279 mm. To further evaluate the vibration absorption performance of the re-entrant auxetic structures, the relative Absorbed Vibration Percentage (AVP) with respect to the solid benchmark was introduced and calculated as Equation (4):

(4) $$AVP=\frac{\left(Vibration\;Amplitude\;of\;solid-Vibration\;Amplitude\;of\;RE\right)}{Vibration\;Amplitude\;of\;Solid}$$

Thus, for the results shown in Figure 4, the AVP is determined to be 60.2% for the PLA specimen with the embedded re-entrant auxetic structure. This approach allows for the quantitative analysis of FIV in the presence of the re-entrant structures under the specified sliding conditions.

### 2.5. Microscopy Analysis

The worn surface morphology of the wear test samples was examined in scanning electron microscopy (SEM) using a Phenom XL Desktop SEM (Thermo Scientific, Waltham, MA, USA). The scanned image provided characteristics of the wear model, taking into account the effect of the post-heat treatment. EDS (energy-dispersive X-ray spectroscopy) was carried out under the same testing machine to detect the element composition of the selected region on the worn surface of the wear sample.

## 3. Results and Discussion

### 3.1. Heat-Treatment Effects on the Mechanical Properties of Printed Polymers

#### 3.1.1. Thermo-Mechanical Properties of Printed Polymers

**Figure 5:**
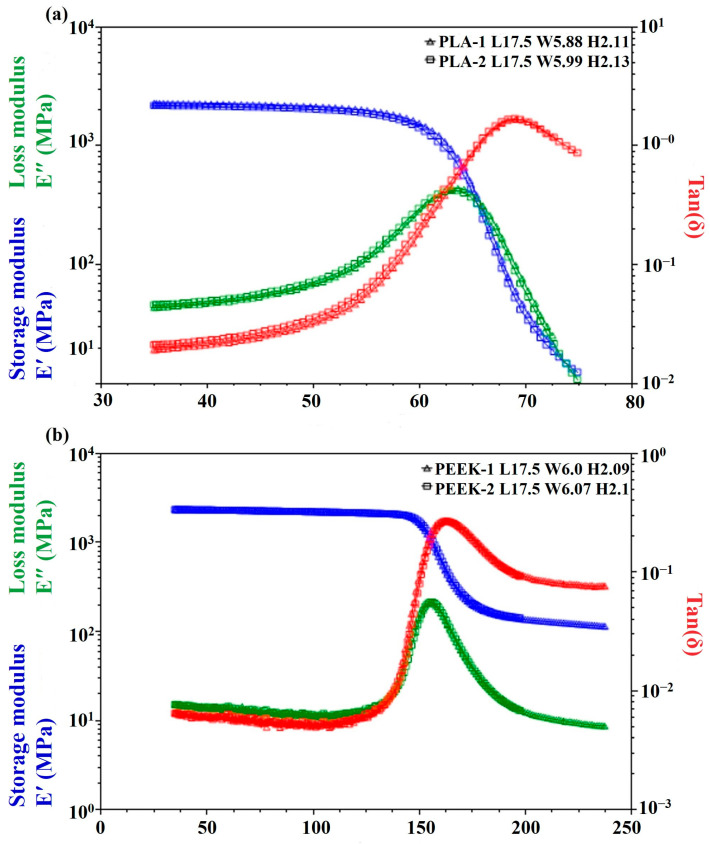
DMA Result for (**a**) PLA and (**b**) PEEK.

DMA tests were conducted twice for both PLA and PEEK, with the dimensions in millimeters of each test specimen indicated in the upper right corner, as shown in Figure 5. Figure 5a presents the DMA results, illustrating the viscoelastic transition behavior of PLA filaments as the temperature increases. The loss modulus exhibits a distinct peak, indicating maximum energy dissipation at the glass transition temperature (T_g_), which corresponds to the tan δ peak at approximately 61.91 °C. Concurrently, the storage modulus shows a pronounced decline near T_g_ due to the onset of large-scale molecular chain mobility. In contrast, PEEK exhibits a significantly higher T_g_ of 151.16 °C. These results are consistent with values reported in the literature [51,52] and provide guidance for selecting the post-heat-treatment temperatures listed in Table 3.

#### 3.1.2. Effects of Post-Heat Treatment

**Figure 6:**
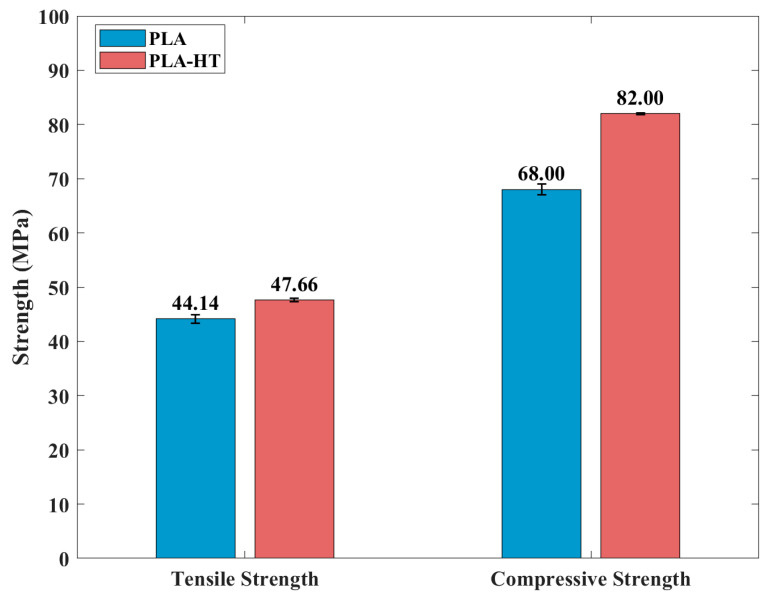
Yield strength and compressive strength of PLA with and without post-heat treatment.

**Figure 7:**
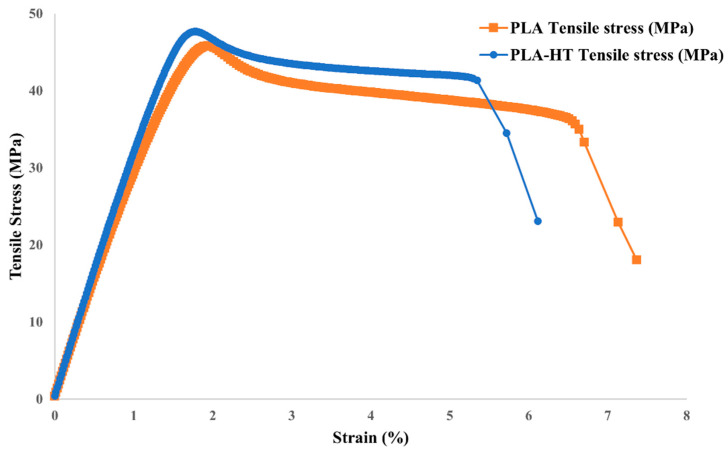
Stress–strain curve of neat PLA with and without post-heat treatment.

Figure 6 compares the mechanical properties of the printed PLA samples before and after post-heat treatment. After baking at 95 °C for 1 h, the strength increased by approximately 10%. For both treated and untreated samples, the printed specimens exhibited higher compressive strength than tensile strength, which can be attributed to void defects introduced during the printing process. Accordingly, the representative tensile stress–strain curves of PLA are shown in Figure 7. It is observed that the elongation at break decreased after heat treatment, indicating reduced ductility, as demonstrated in Figure 7. This observation is consistent with previous studies, which indicated that the heat treatment of FDM-printed PLA influences the degree of crystallinity and layer bonding situation [53]. The process enhances the chain mobility of the amorphous regions, facilitating the crystallization process, lamellar thickening, and promoting the interlayer diffusion. As a result, the collective effects contribute to the enhancement in the tensile strength and material stiffness [42]. However, these studies also highlighted the trade-off in mechanical properties. Excessive crystallization can disrupt the uniformity of the amorphous phase and introduce microstructural heterogeneities, which result in stress concentration sites and decreased ductility [53]. This explains the results observed in Figure 7.

**Figure 8:**
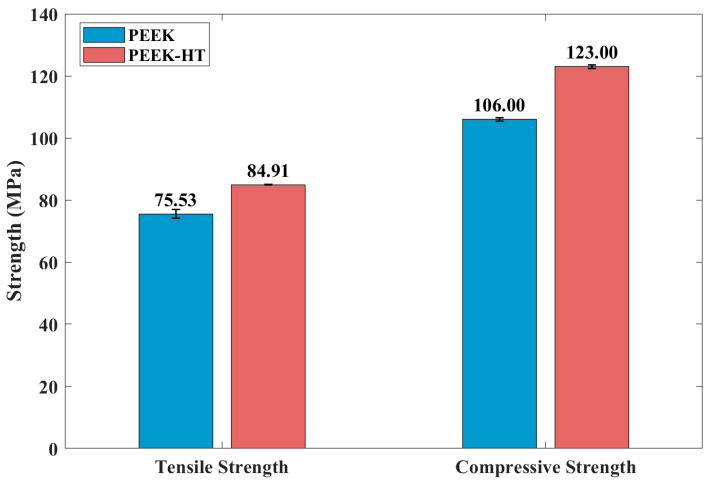
Yield strength and compressive strength of PEEK with and without post-heat treatment.

**Figure 9:**
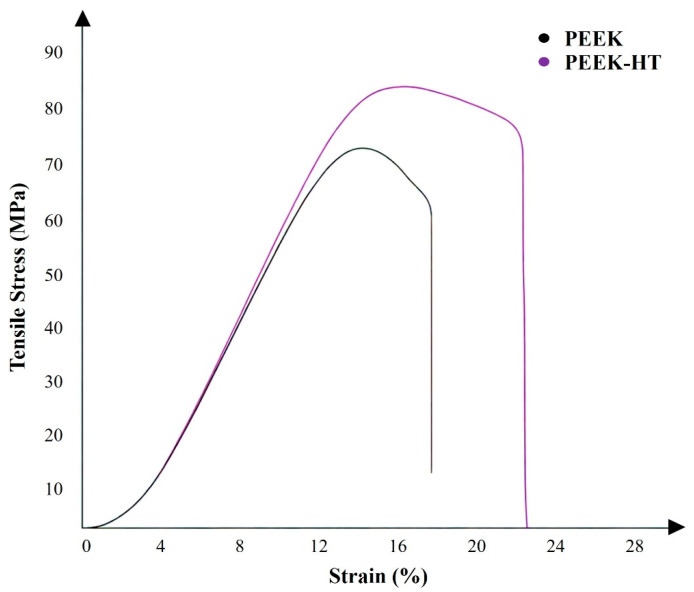
Stress–strain curve of neat PEEK with and without post-heat treatment.

For PEEK, the mechanical strength was also enhanced after post-heat treatment, as shown in Figure 8. The characteristic tensile stress–strain curves of the untreated and heat-treated PEEK materials are presented in Figure 9. It was observed that ductility increased following heat treatment. In general, improvements in mechanical properties such as strength and modulus can be attributed to an increased degree of crystallinity and enhanced interlayer bonding [30,47,54]. However, the effect of heat treatment on the ductility of PEEK is more complex. As reported by Yang et al. [30], the ductility of printed PEEK initially decreased as crystallinity increased from 16% to 18% but subsequently increased with further increases in crystallinity from 19% to 21%.

**Figure 10:**
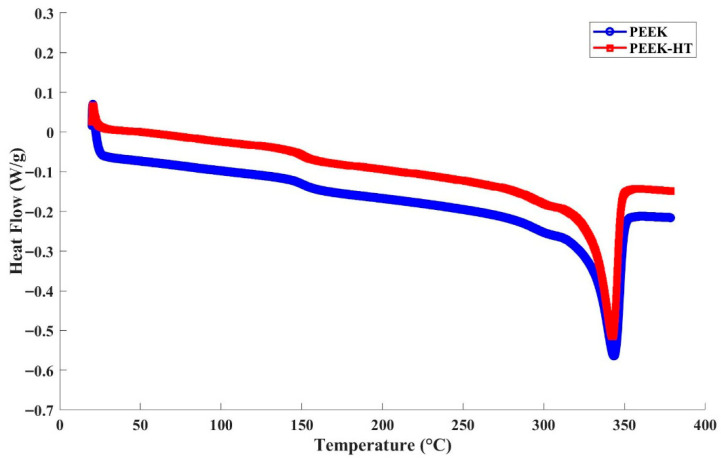
The DSC result of PEEK with and without post-heat treatment.

To quantify the crystallinity of the printed PEEK materials, DSC tests were conducted, and the results are summarized in Figure 10. Using Equation (1), the degree of crystallinity of standard PEEK was calculated to be 21.8%, which increased to 24.0% after post-heat treatment, as listed in Table 3. These results are consistent with the findings reported by Yang et al. [30], i.e., ductility increases with crystallinity when the crystallinity is higher than 19%. Nevertheless, it should be noted that, in addition to crystallinity, other factors such as residual stress, internal defects, and structural distortion induced by heat treatment may also influence the elongation at break of printed PEEK. In particular, high residual stress can be introduced during the printing process due to the relatively high processing temperature. Annealing can effectively relieve this residual stress, which, together with increased crystallinity and improved interlayer bonding, may account for the observed enhancement in ductility of the printed PEEK materials.

### 3.2. Heat Treatment Effects on Friction and Wear Behavior

#### 3.2.1. Friction and Wear Behavior of Printed PLA

**Figure 11:**
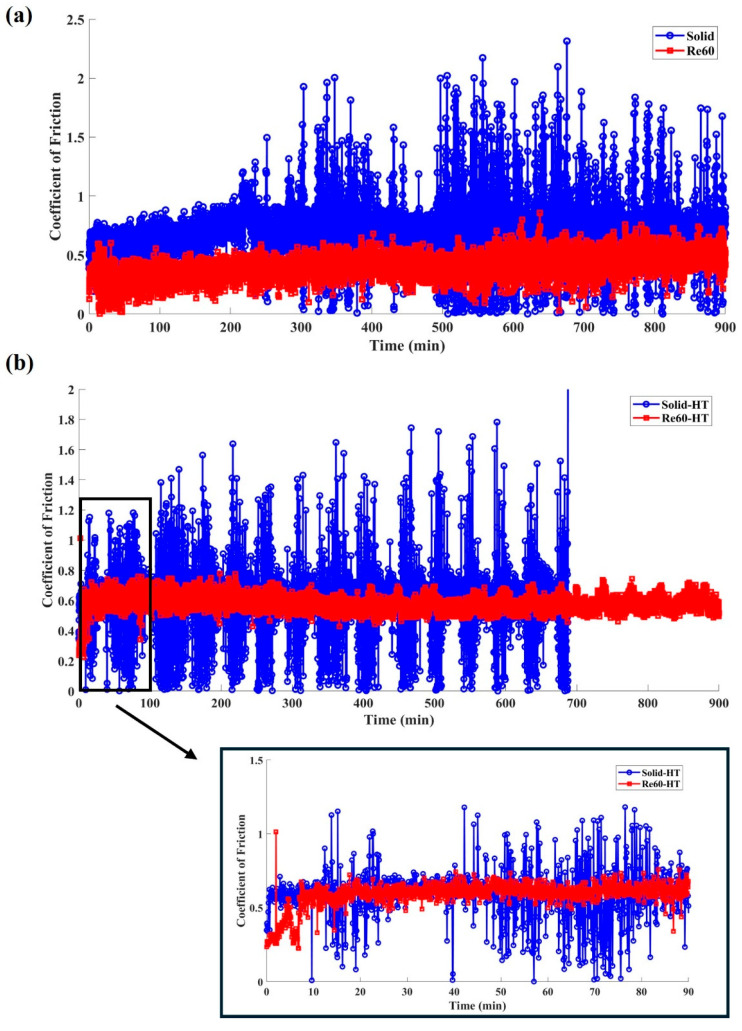
Coefficient of friction of PLA samples with (Re60) and without re-entrant (solid) structure tested under 5 N: (**a**) before and (**b**) after heat treatment.

Figure 11 compares the typical friction responses of the printed PLA samples before and after heat treatment. All tests were conducted under an applied normal load of 5 N and a constant disk rotation speed of 120 rpm. For the untreated solid benchmark sample (cf. Figure 2c), the coefficient of friction (CoF) exhibited a slight increase during the initial stage, up to approximately 200 min. This behavior is likely associated with an increase in the real contact area and contact temperature resulting from frictional heating. Beyond this stage, significant fluctuations in the CoF were observed. After post-heat treatment, the solid benchmark sample showed an early and abrupt increase in CoF, with pronounced fluctuations initiating at approximately 48 min, as shown in Figure 11b. As sliding continued, these oscillations became dominant, and the test was terminated at 687 min when the CoF exceeded the upper threshold limit. The severe CoF fluctuations can be attributed to the increased stiffness and brittleness of PLA after post-heat treatment. This behavior hinders the formation of a stable and uniform transfer film on the steel counterpart, thereby leading to increased CoF fluctuations during sliding [55].

**Figure 12:**
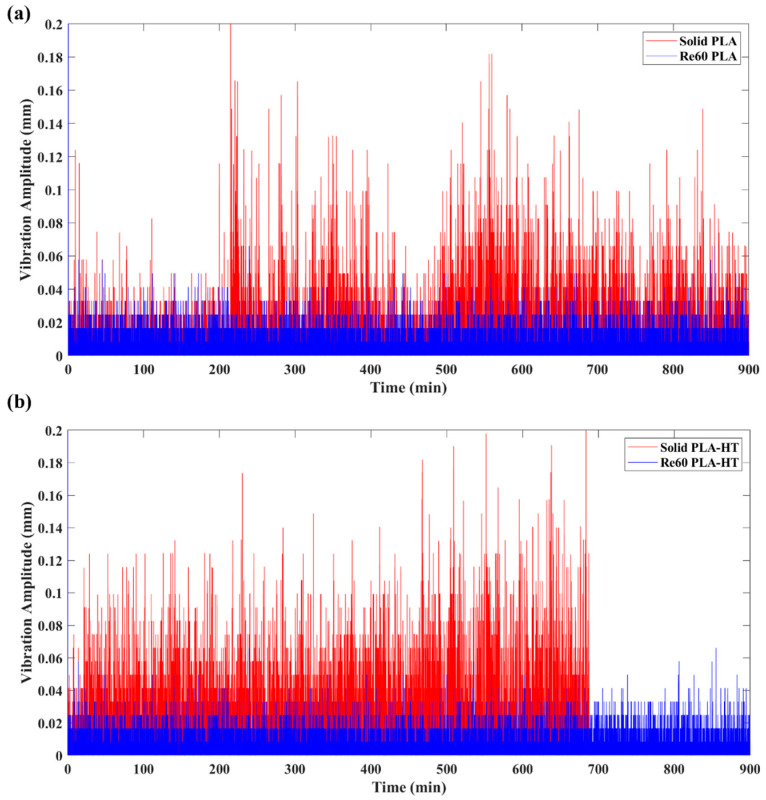
Vibration amplitude of PLA samples with (Re60) and without re-entrant (solid) structure tested under 5 N: (**a**) before and (**b**) after heat treatment.

The pronounced fluctuations in friction observed above are generally associated with vibrations, namely friction-induced vibration (FIV), in the sliding system [56,57]. In this study, the vibration amplitude was calculated using Equation (3) based on the vertical displacement of the sliding samples captured by an LVDT sensor. Figure 12 illustrates the effects of post-heat treatment on the FIV behavior of the printed materials. It is evident that samples with embedded re-entrant structures, both heat-treated and untreated, exhibited significantly reduced FIV compared with the solid benchmark sample. To quantitatively evaluate the vibration absorption performance and assess the effect of post-heat treatment on FIV, the relative absorbed vibration percentage (AVP) with respect to the solid benchmark sample was calculated based on Equation (4) above.

**Figure 13:**
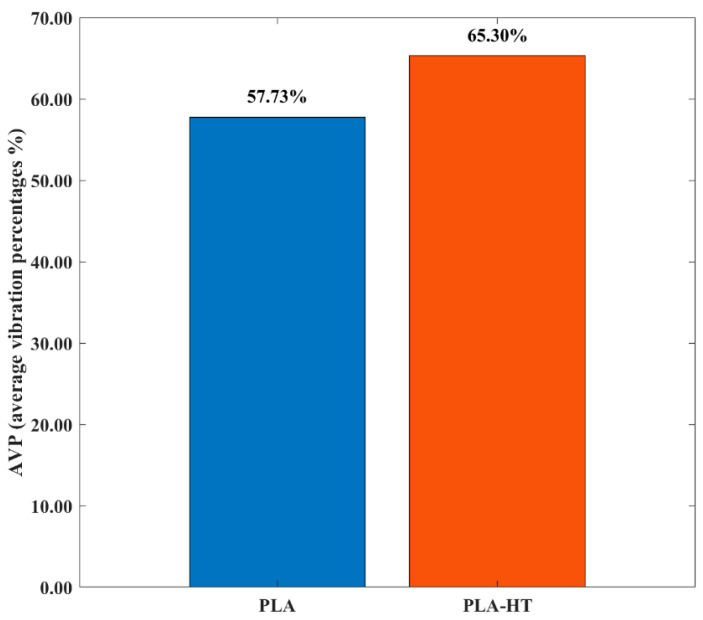
AVP of PLA samples with the re-entrant structures before (PLA) and after heat-treated PLA (PLA-HT).

Figure 13 presents the AVP results for PLA samples with the re-entrant structures before and after heat-treated PLA (PLA-HT). It can be seen that FIV was effectively mitigated by the embedded re-entrant auxetic structures due to their inherent vibration absorption capabilities [58,59]. Furthermore, the heat-treated re-entrant wear model exhibited a more pronounced vibration absorption capacity, reaching 65.30%. While post-heat treatment enhances the mechanical performance of PLA, the associated increase in the degree of crystallinity partially transforms the amorphous regions into crystalline lamellae, leading to higher stiffness and increased structural rigidity [60]. This improvement in stiffness enhances the energy absorption capability of the re-entrant structures [61,62], thereby contributing to the observed reduction in FIV [40].

**Figure 14:**
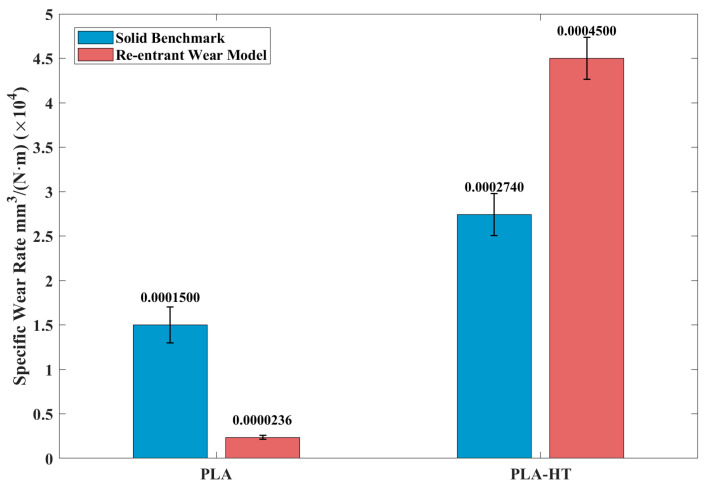
The effects of post-heat treatment on printed PLA samples before and after heat treatment were tested under 5 N.

**Figure 15:**
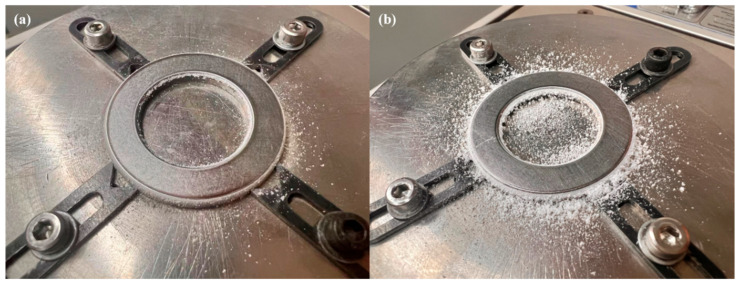
Smearing wear debris on the disk after the test of the heat-treated sample printed in PLA: (**a**) without and (**b**) with a re-entrant structure.

Figure 14 summarizes the specific wear rate (SWR) of the printed PLA samples. For all heat-treated wear specimens, the SWR generally increased. Notably, for samples with embedded re-entrant structures, the SWR increased significantly after the heat treatment, from 0.0000236 ${mm}^{3}/(N\cdot m)$ to 0.0004500 ${mm}^{3}/\left(N\cdot m\right)$. This substantial increase in wear is also evidenced by the markedly greater amount of wear debris distributed around the steel counterpart after testing, as shown in Figure 15.

#### 3.2.2. Friction and Wear Behavior of Printed PEEK

**Figure 16:**
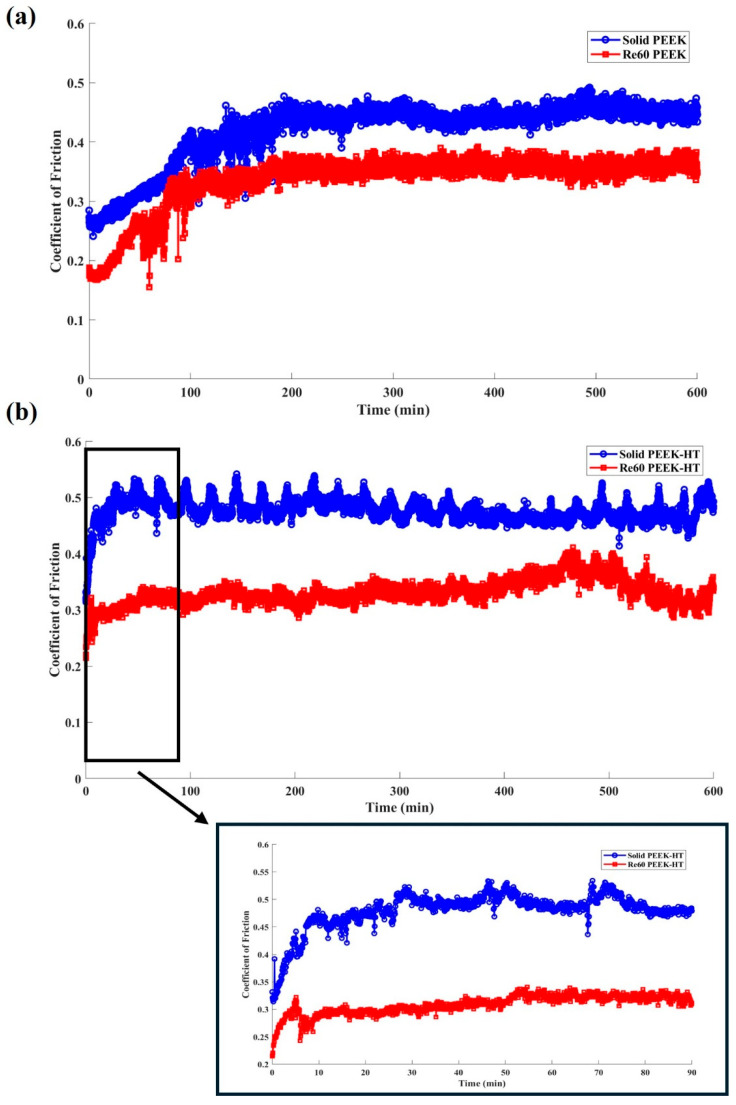
Coefficient of friction of PEEK samples with (Re60) and without a re-entrant (solid) structure tested under 25 N: (**a**) before and (**b**) after heat treatment.

Figure 16 compares the typical friction responses of printed PEEK materials with and without post-heat treatment. All tests were conducted under an applied normal load of 25 N for a total testing duration of 600 min. As shown in Figure 16a, the CoF slightly increased during the running-in stage, which lasted approximately 200 min. This behavior is similar to that observed for PLA. Owing to the excellent mechanical properties and high wear resistance of PEEK, the untreated solid benchmark sample did not exhibit significant oscillations, like those observed for PLA during the later stages of sliding. After post-heat treatment, the running-in stage was shortened to approximately 50 min, as shown in Figure 16b. This behavior is likely due to the increased degree of crystallinity [63] and ductility of the material, which promotes faster formation of a stable transfer film [64]. Improved surface integrity and reduced interfacial shear consequently enhance the stability of the CoF. Moreover, samples with embedded re-entrant structures exhibited reduced CoF under both heat-treated and untreated conditions.

**Figure 17:**
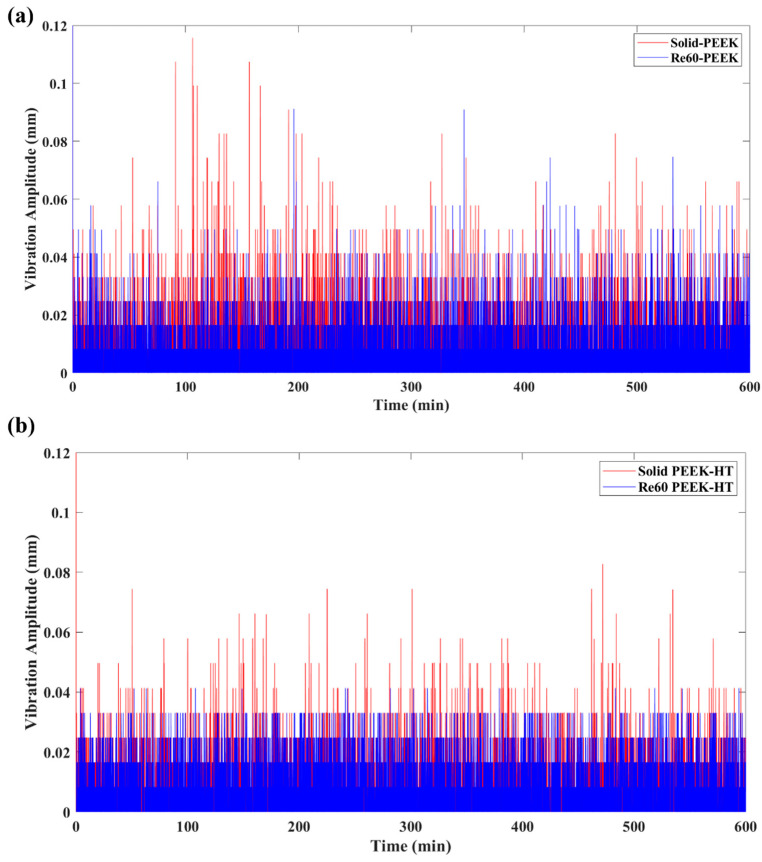
Vibration amplitude of PEEK samples with (Re60) and without a re-entrant (solid) structure tested under 25 N: (**a**) before and (**b**) after heat treatment.

Figure 17 presents the vibration amplitude of the PEEK wear models before and after heat treatment. Owing to the superior mechanical properties of PEEK, the solid benchmark sample exhibited relatively low FIV, with a vibration amplitude of 0.00136 mm. In contrast, the sample with embedded re-entrant auxetic structures further suppressed FIV, reducing the amplitude to 0.00109 mm. This behavior is consistent with the results observed for heat-treated PLA, as discussed above, and aligns with the conclusions reported in our previous study [40].

**Figure 18:**
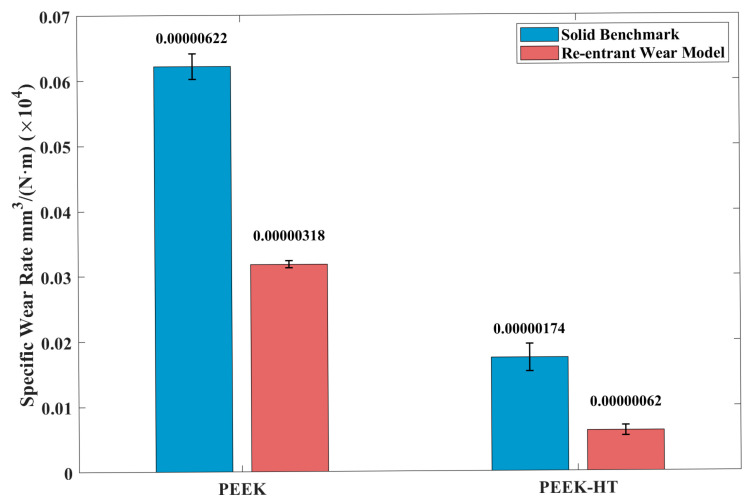
The effects of post-heat treatment on printed PEEK samples before and after heat treatment were tested under 25 N.

Post-heat treatment had a detrimental effect on the PLA wear samples, as shown in Figure 14, whereas the PEEK samples exhibited the opposite trend. As seen in Figure 18, the SWR of heat-treated PEEK wear models generally decreased. Notably, the re-entrant wear model showed a significant reduction, from 0.00000318 ${mm}^{3}/(N\cdot m)$ to 0.00000062 ${mm}^{3}/\left(N\cdot m\right)$. To further understand the underlying wear mechanisms responsible for these opposite SWR trends in PLA and PEEK induced by post-heat treatment, the worn surfaces were examined using SEM and EDS, as discussed in the following section.

#### 3.2.3. Discussion

**Figure 19:**
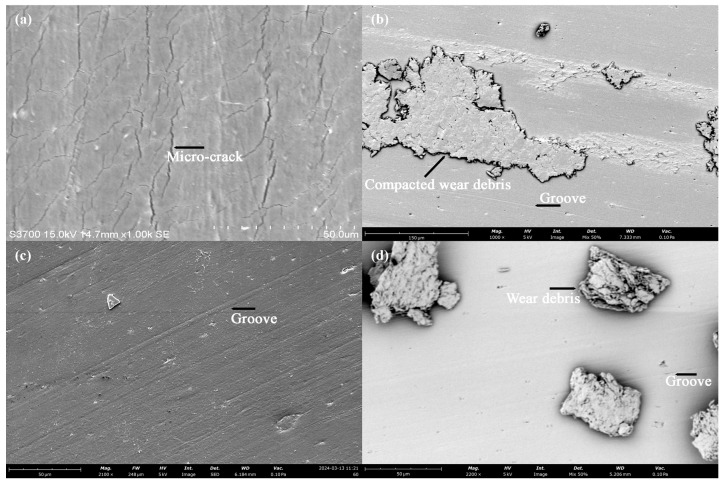
SEM images of the worn surfaces of the PLA sample tested at 5 N: (**a**) solid benchmark sample before heat treatment, (**b**) solid benchmark sample after heat treatment, (**c**) with a re-entrant structure before heat treatment, and (**d**) with a re-entrant structure after heat treatment.

Figure 19 presents the SEM images of the worn surfaces of printed PLA before and after post-heat treatment. In Figure 19a, numerous micro-cracks are observed on the worn surface of the PLA sample without re-entrant structures. As indicated in Figure 11a and Figure 12a, severe friction oscillations and vibrations occurred during the sliding process of the sample. The conditions likely resulted in surface fatigue due to alternating compressive and tensile stresses, which consequently promoted the formation of micro-cracks on the worn surface [65,66]. With the embedded re-entrant structure, however, both friction oscillations and FIV were effectively mitigated (cf. Figure 11a). As a result, no observable cracks were detected on the worn surface, as shown in Figure 19c. Instead, grooves aligned along the sliding direction were visible, indicating a mild abrasive wear process. Consequently, the wear rate was significantly reduced, as demonstrated in Figure 14.

After the post-heat treatment, the increased brittleness of the material promoted the formation of powder-shaped debris during the wear process, as shown in Figure 15. For the solid specimen, severe FIV occurred, as illustrated in Figure 11b. In this case, the intensive FIV in the vertical direction repeatedly impacted the loosened wear debris, resulting in compacted debris on the worn surface, as shown in Figure 19b. The compacted debris may have acted as a cushion, reducing surface cracks on the worn surface. With the embedded re-entrant structures, FIV was effectively reduced. Consequently, larger-sized particulate wear debris was observed on the worn surface, as shown in Figure 19d. These larger debris particles likely acted as a three-body abrasion medium, contributing to increased wear loss of the sample. As a result, the highest wear loss was observed for the sample with the embedded re-entrant structures.

**Figure 20:**
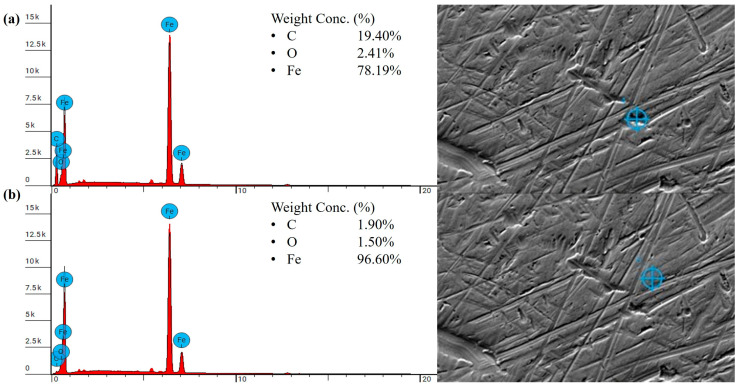
EDS result of the steel counterpart tested against the heat-treated PLA solid benchmark sample tested under 5 N: (**a**) isolated dark regions, (**b**) mostly bare steel regions.

**Figure 21:**
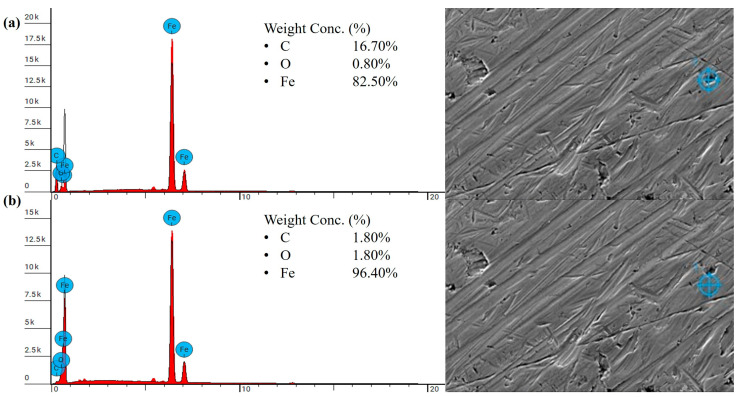
EDS result of the steel counterpart tested against the heat-treated PLA sample with a re-entrant structure under 5 N: (**a**) isolated dark regions, (**b**) mostly bare steel regions.

The SEM and EDS analyses were performed on the steel counterpart sliding against the heat-treated PLA samples. The results indicate that no effective transfer film was formed on the steel counterpart, which explains the high wear loss observed in the heat-treated samples. The elemental composition of the small, isolated dark regions on the steel counterpart showed an increase in carbon content, confirming the presence of polymeric debris. The carbon content in these regions increased to 19.40 wt.% compared to the mostly bare steel, which had a carbon content of 1.90 wt.%, as shown in Figure 20. For the heat-treated PLA sample with the re-entrant structure, the EDS results revealed a similar trend in carbon content on the steel counterpart. The darker regions containing polymer debris exhibited an increase in carbon content to 16.70 wt.% compared to the bare steel-dominated regions, which had a carbon content of 1.80 wt.%, as shown in Figure 21.

**Figure 22:**
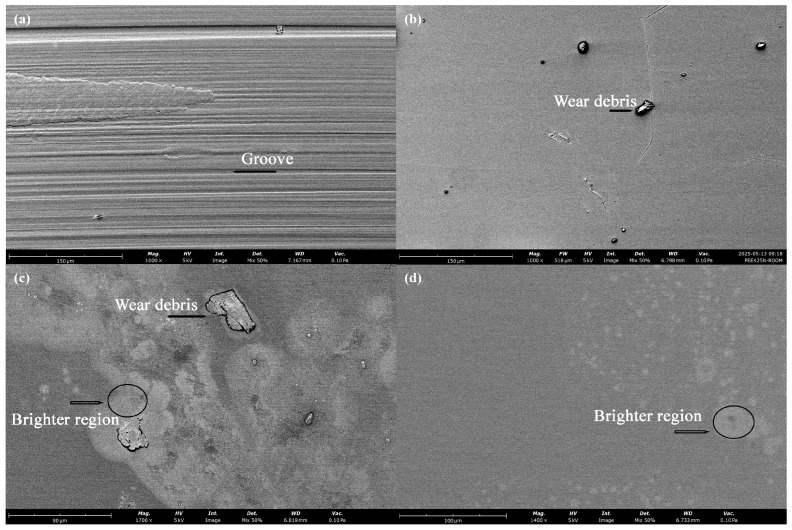
SEM images of the worn surfaces of the PEEK sample tested at 25 N: (**a**) solid benchmark sample before heat treatment, (**b**) with a re-entrant structure before heat treatment, (**c**) solid benchmark sample after heat treatment, and (**d**) with a re-entrant structure after heat treatment.

For PEEK material, owing to its favorable mechanical property profile, all PEEK samples exhibited reduced CoF fluctuations and FIV. The worn surface condition of the solid benchmark was smoother, displaying uniform grooves along the sliding direction, as shown in Figure 22a. This indicates a mild abrasion wear mechanism. With the incorporation of the embedded re-entrant structure, the CoF further decreased with even lower FIV, and the worn surface became smoother (Figure 22b). This improvement was accompanied by a reduction in SWR (Figure 18).

**Figure 23:**
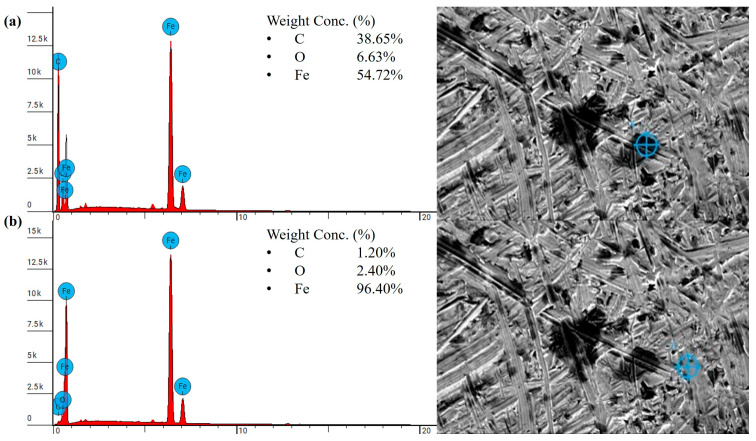
EDS result of the steel counterpart against the heat-treated PEEK solid benchmark sample tested under 25 N: (**a**) dark regions, (**b**) steel-dominated regions.

**Figure 24:**
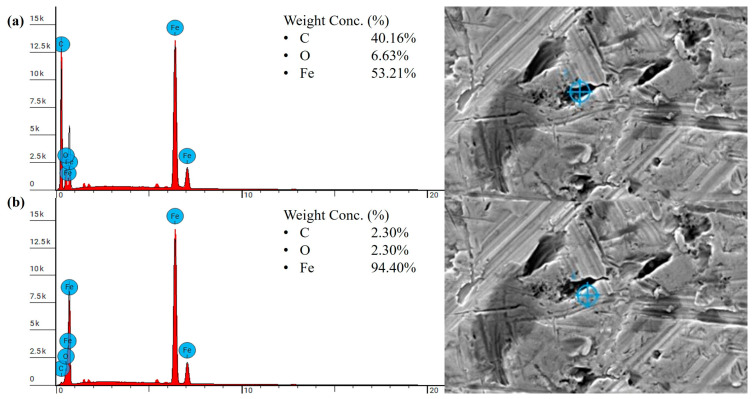
EDS result of the steel counterpart tested against the heat-treated PEEK sample with a re-entrant structure tested under 25 N: (**a**) dark regions, (**b**) steel-dominated regions.

After heat treatment, the strength and ductility of the material further improved, resulting in a smoother worn surface, as shown in Figure 22c,d. In general, with increased ductility, the polymer tends to undergo plastic deformation rather than brittle fracture, which typically facilitates the formation of a stable protective transfer film [67]. SEM and EDS analyses confirmed that a greater number of patched transfer film layers were observed on the steel counterpart. As shown in Figure 23, the dark regions exhibited significantly higher carbon content, reaching up to 38.65 wt.%, along with a notable reduction in Fe content to 54.72%, compared to the steel-dominated regions with the Fe content of 96.40 wt.%. The higher carbon content of the polymer patches on the steel, nearly twice that of PLA, indicates the formation of a comparatively thicker and more effective transfer film. Notably, unlike the extensive distribution of wear debris observed on the heat-treated re-entrant wear model printed in PLA (Figure 19d), no grooves or wear debris were detected on the worn surface of the re-entrant wear model made of PEEK after heat treatment, as shown in Figure 22d. This is likely due to the increased strength and ductility. The higher carbon content of up to 40.16 wt.% observed in the darker regions from the EDS analysis (Figure 24) suggests the formation of an effective transfer film compared to the heat-treated solid benchmark. Furthermore, both heat-treated wear samples displayed slightly brighter regions occupying a notable portion of the scanned area, as seen in Figure 22c,d. These regions correspond to dense lamellar structures with a high load-bearing capacity [68]. As a result, the heat-treated sample with embedded re-entrant structures exhibited the highest wear resistance.

## 4. Conclusions

This study investigated the effects of post-heat treatment on the mechanical and tribological properties of PLA and PEEK materials and structures fabricated using FDM technology. In particular, the tribological performance of printed specimens with and without embedded re-entrant auxetic structures was comparatively evaluated. The following conclusions can be drawn:

1. The proposed heat treatment resulted in higher degrees of crystallinity and, consequently, improved strength for both printed materials. For PLA, heat treatment increased the tensile strength from 44.14 MPa to 47.66 MPa, while the compressive strength improved from 68 MPa to 82 MPa. A similar trend was observed for heat-treated PEEK, with tensile strength increasing from 75.53 MPa to 84.91 MPa and compressive strength improving from 106 MPa to 123 MPa. Nevertheless, post-heat treatment reduced the ductility of PLA while enhancing the ductility of PEEK.
2. Under all testing conditions, the embedded re-entrant auxetic structures contributed to a lower coefficient of friction (CoF) and reduced friction-induced vibration (FIV) owing to their high energy absorption capacity. The results indicate that post-heat treatment can deteriorate the CoF behavior of solid benchmark PLA specimens, as evidenced by the earlier onset and increased intensity of CoF fluctuations. In contrast, specimens incorporating re-entrant auxetic structures exhibited a comparatively more stable CoF after heat treatment. Furthermore, the enhanced stiffness of the embedded re-entrant structures resulting from post-heat treatment led to a more pronounced mitigation of FIV in the printed specimens.
3. Post-heat treatment exhibited contrasting effects on the specific wear rate (SWR) of the two printed materials. For PLA, the treatment increased material brittleness, leading to a higher SWR. Specifically, after post-heat treatment, the SWR of the solid benchmark PLA increased from 1.5 × 10^−4^${mm}^{3}/(N\cdot m)$ to 2.74 × 10^−4^${mm}^{3}/(N\cdot m)$. For specimens incorporating re-entrant structures, the wear rate was generally reduced compared with solid ones due to the mitigation of friction-induced vibrations (FIV). Nevertheless, post-heat treatment also resulted in an increase in SWR of PLA samples with the re-entrant structure from 2.36 × 10^−5^${mm}^{3}/(N\cdot m)$ to 4.5 × 10^−4^${mm}^{3}/(N\cdot m)$. This behavior is attributed to the increased brittleness of heat-treated PLA, which promoted the formation of powder-like wear debris and, consequently, higher wear loss despite the improved strength. In contrast, for PEEK, post-heat treatment enhanced both strength and ductility, leading to a significant improvement in wear resistance. Specifically, the SWR of the solid benchmark PEEK decreased from 6.2 × 10^−6^ ${mm}^{3}/(N\cdot m)\;$ to 1.74 × 10^−6^ ${mm}^{3}/(N\cdot m)\;$ after heat treatment. Similarly, the re-entrant structured specimens exhibited a reduction in SWR from 3.18 × 10^−6^${mm}^{3}/(N\cdot m)\;$ to 6.2 × 10^−7^ ${mm}^{3}/(N\cdot m)$.

Finally, it is worth noting that the present work focuses on the distinctive effects of post-heat treatment on the tribological properties of different polymers, namely PLA and PEEK. The annealing process was selected as a commonly used and cost-efficient post-processing technique for 3D-printed parts. Nevertheless, as summarized in the literature review, other post-treatment methods, such as chemical smoothing [26] and the ironing process [27], are also available. Future work will be carried out to further explore additional effective treatment methods to achieve improved tribological properties under different sliding conditions. In particular, the combination of multiple treatment methods may allow further tailoring and optimization of the property profiles of 3D-printed polymer components for different engineering applications.

## Figures and Tables

**Table 1:** Printing parameters of filaments.

Filament	Nozzle Temperature (°C)	Bed Temperature (°C)	Chamber Temperature (°C)
Neat PLA	210	60	Room
Neat PEEK	400	145	90

**Table 3:** Post-heat treatment settings.

Sample	Temperature and Duration
Neat PLA	Increase from room temperature to 95 °C at a rate of 65 °C/h Holding 95 °C for 1 h Cool down to room temperature
Neat PEEK	Increase from room temperature to 120 °C at a rate of 60 °C/h Holding 120 °C for 2 h Increase from 120 °C to 200 °C at a rate of 80 °C/h Holding 200 °C for 30 min Increase from 200 °C to 220 °C at a rate of 60 °C/h Holding 220 °C for 3 h Cool down to room temperature

**Table 2:** DSC settings for PEEK and PEEK-HT.

Sample	Temperature and Duration
PEEK and PEEK-HT	1. Holding room temperature at 20 °C for 1 min
	2. Increase from 20 °C to 380 °C in ramp 10 °C/min
	3. Holding 380 °C for 1 min
	4. Decrease from 380 °C to 20 °C in ramp 5 °C/min
	5. Holding room temperature at 20 °C for 1 min

## Data Availability

The original contribution presented in this paper is included in the article. Further inquiries can be directed to the corresponding author.
